# Management of an Implant-Supported Fixed Partial Denture in the Esthetic Zone in a Patient With a Very Limited Mouth Opening: A Case Report

**DOI:** 10.7759/cureus.57107

**Published:** 2024-03-28

**Authors:** Frederic Silvestri, Thomas Stephan, Charlotte Mansuy, Chloë Mense

**Affiliations:** 1 Implantology, Aix-Marseille University, School of Dental Medicine, Assistance Publique des Hôpitaux de Marseille (APHM), Marseille, FRA

**Keywords:** case report, limited opening mouth, maxilla, temporary dental prosthesis, tissue conditioning, esthetics, dental implants

## Abstract

In the maxillary anterior area, the esthetic integration of prosthetic restorations is a challenge, particularly for screw-retained implant prostheses. This case report presents the management and clinical outcome of an old partial edentulous maxillary jaw in an esthetic zone in a young patient with a very limited mouth opening. This patient was rehabilitated with an implant screw-retained fixed partial denture (FPD) using both digital and conventional techniques.

## Introduction

In the maxillary anterior area, the esthetic integration of prosthetic restorations is a challenge, particularly for screw-retained implant prostheses. Objective assessment of success can be based on the pink and white esthetic scores, which evaluate both the prosthetic restoration and the surrounding soft tissue [1,2]. In the case of an old partially edentulous jaw, a significant part of this challenge is related to the reconstruction of the lost papillae. Achieving functional and esthetic outcomes requires diagnostic skills and high accuracy in the positioning of implants in relation to the prosthetic plan. In daily practice, digital technologies and guided surgery (GS) can help achieve this goal and are of great importance in complex clinical situations such as limited opening mouth.

This case report presents the management and clinical outcome of an old partially edentulous maxillary jaw in an esthetic zone in a young patient with a very limited mouth opening. This patient was rehabilitated with an implant screw-retained fixed partial denture (FPD) using both digital and conventional techniques. The case was followed up for 18 months.

## Case presentation

**Figure 1:**
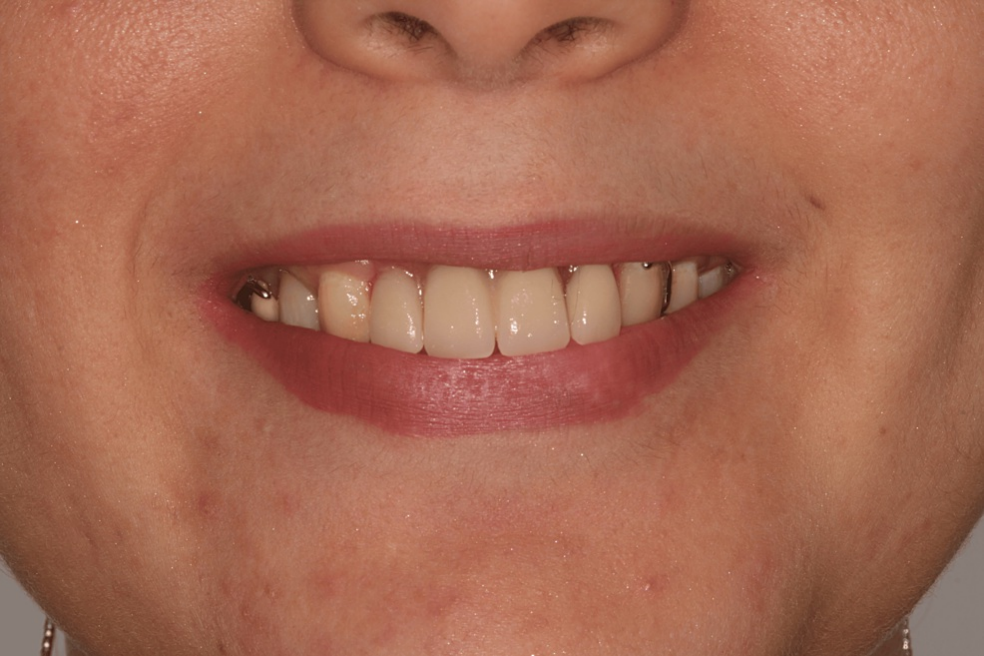
Initial situation with removable partial denture

**Figure 2:**
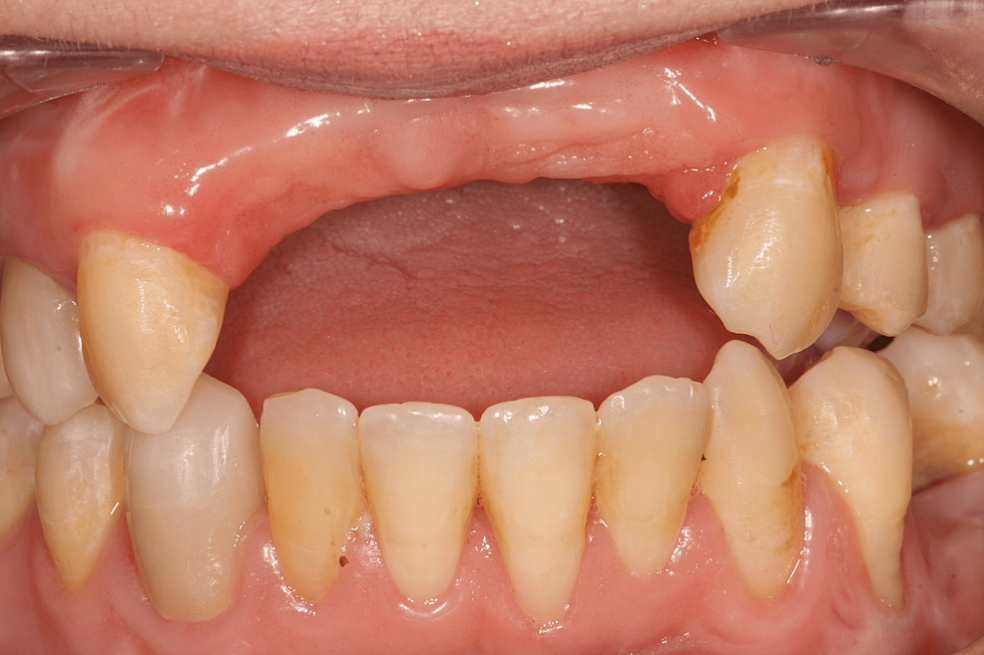
Initial situation with removable denture

**Figure 3:**
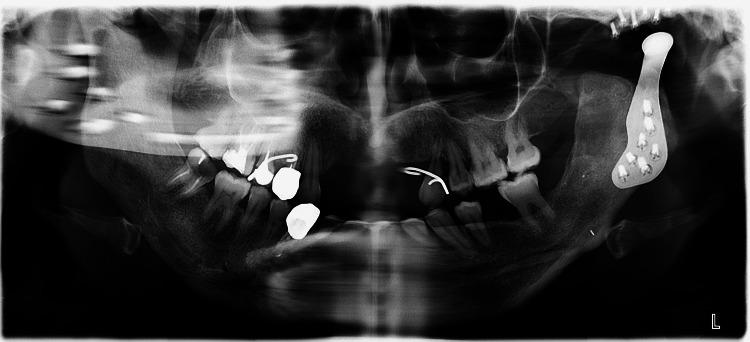
Panoramic radiograph of the initial situation

A 25-year-old female came for her first visit to the Department of Implantology, Timone University Hospital, Marseille, France. Fourteen years ago, the patient suffered a maxillofacial trauma following a road accident, which resulted in the loss of her maxillary incisors, and several facial fractures including a mandibular trifocal fracture. Later, she underwent an ankylosis of the left temporomandibular joint (TMJ). The left temporomandibular joint has been replaced with a prosthesis, and the missing maxillary anterior teeth have been restored with a removable partial denture (RPD) for 13 years (Figures 1-3).

In 2018, the patient underwent a LeFort 1 maxillary orthognathic surgery. She did not smoke tobacco or drink alcohol. Currently, she is not taking any medication and has no known allergies. She wanted to replace the removable partial denture with a fixed denture.

Exobuccal examination showed an asymmetry in the lower lip, which is slightly fleshier on the left side (Figure 1). Endobuccal examination revealed a very limited mouth opening (25 mm). Long-term wear of RPD has led to total loss of the papillae in the edentulous area (Figure 2). RPD's artificial teeth showed correct dimensions. There was a small vertical loss of supporting tissue in the area of missing teeth #21 and #22 (Figure 2).

The panoramic radiograph showed a complete TMJ prosthesis in two parts (Figure 3). Cone beam computed tomography (CBCT) (PlanMeca ProMax 3D®, Helsinki, Finland) has highlighted an external root resorption on tooth #23, indicating extraction.

The patient did not want to undergo any grafting procedure. After discussion and explanation of existing treatment options, the decision was made to proceed with a partial screw-retained implant-supported prosthesis without vertical bone augmentation surgery. The treatment sequence will consist of the placement of three implants (#12, #22, and #23) on the same day as the extraction of tooth #23. Immediate loading will consist of the use of a temporary screw-retained prosthesis. After osseointegration, a definitive screw-retained FPD will be delivered [3].

**Figure 4:**
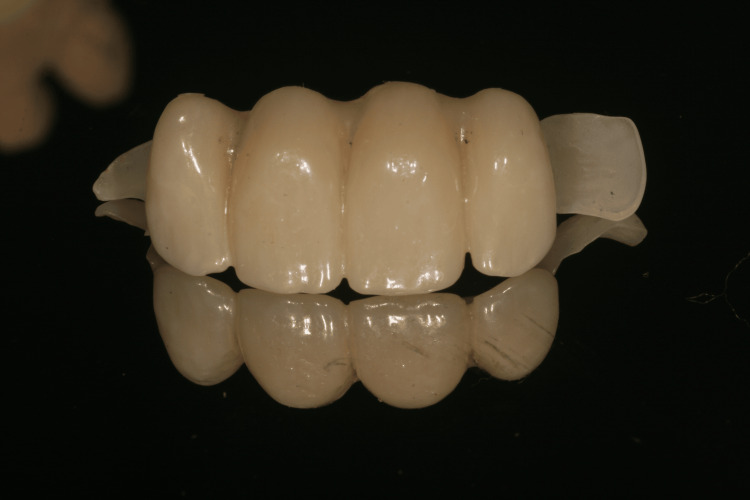
Acrylic removable prosthetic project

**Figure 5:**
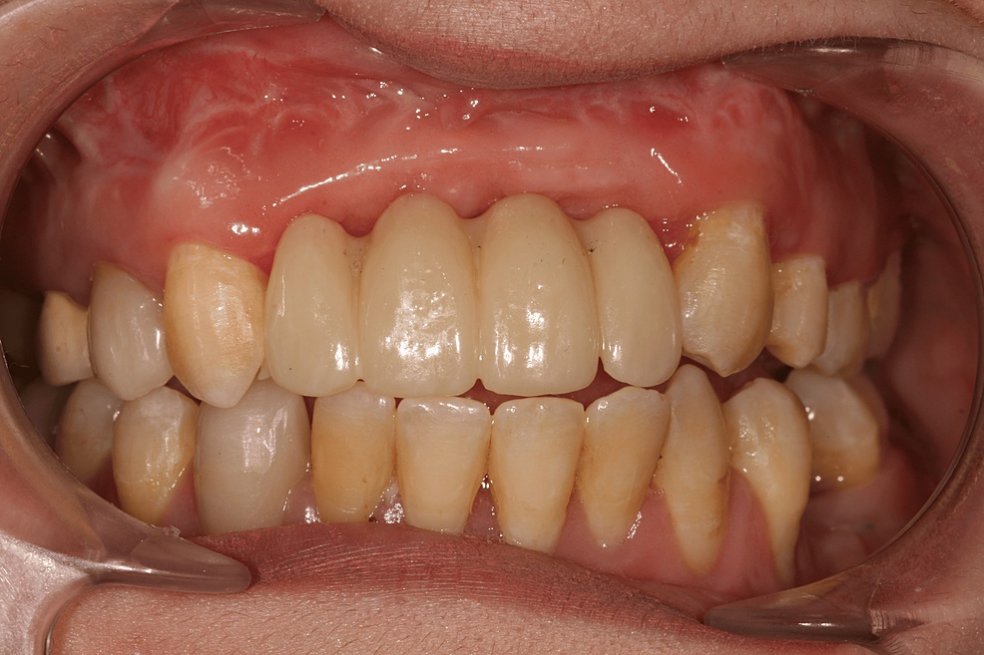
Prosthetic project try-in

The planning phase was the first stage of treatment, which began with a functional and esthetic plan. It took into account the patient's wishes and clinical considerations. First, an acrylic resin removable partial prosthesis (Telio CAD®, Ivoclar Vivadent AG, Schaan, Liechtenstein) stabilized by two wings was tried and met the patient's satisfaction (shape, dimensions, and color). Obviously, periodontal therapy has been carried out before the implants were placed to ensure periodontal health and increase the chances of successful implant surgery (Figures 4, 5). Digital planning began with intraoral scanning (TRIOS 3®, 3Shape A/S, Copenhagen, Denmark). 

**Figure 6:**
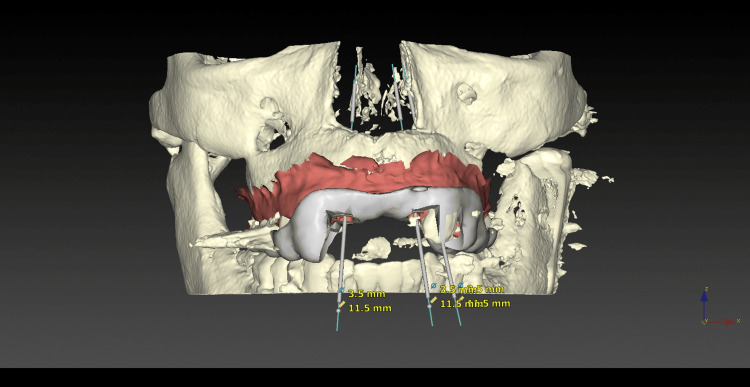
Prosthetically guided implant planning

**Figure 7:**
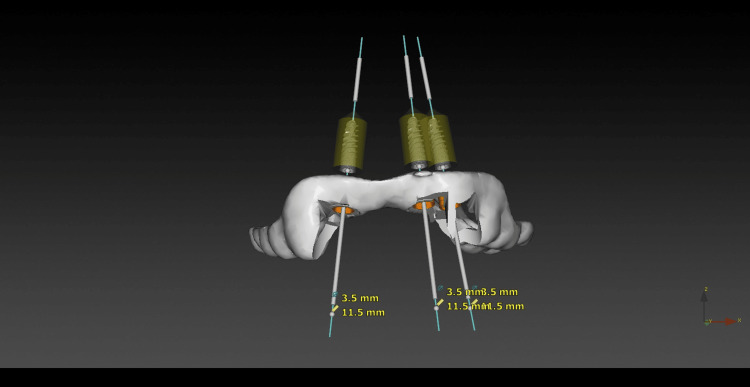
Virtual implant planning and surgical guide

**Figure 8:**
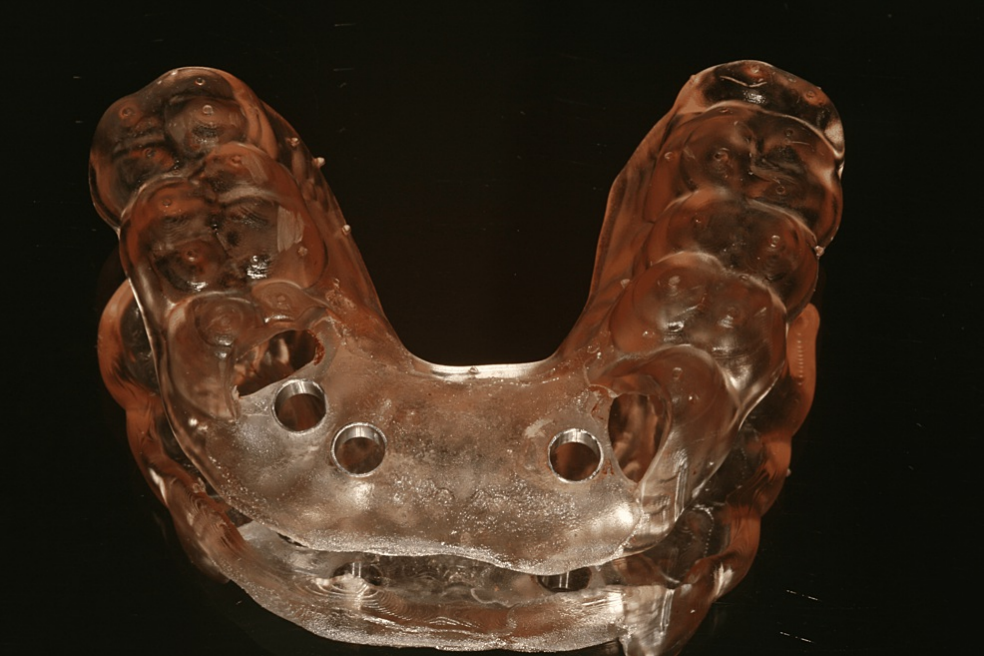
Tooth-supported surgical guide

Both the Digital Imaging and Communication in Medicine (DICOM) file (CBCT) and Standard Tessellation Language (STL) file were uploaded into the implant planning software (DTX Studio®, version 3.6.4.2, Nobel Biocare AB, Göteborg, Sweden). A virtual prosthetic project was also created using information collected during the first trial. Implant planning will therefore be prosthetically guided. Implant design and dimensions have been chosen according to the bone volume available and willingness to perform an immediate loading. A fully guided tooth-supported surgical guide was automatically drawn by the planning software and printed by the technician (Figures 6-8).

**Figure 9:**
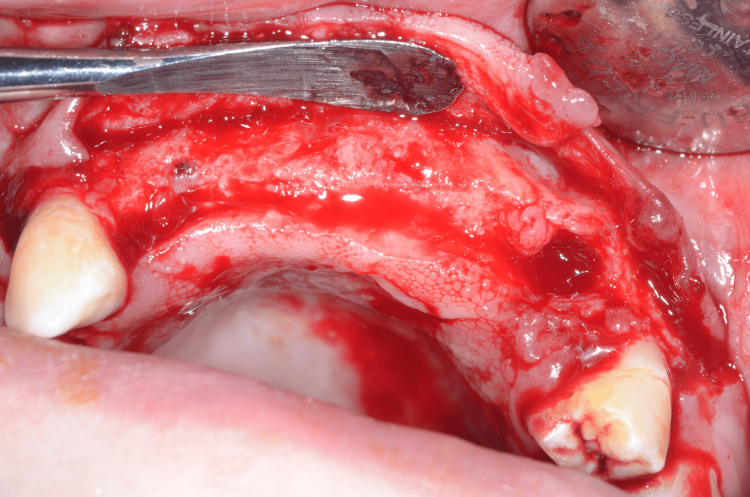
Full-thickness flap with an incision as palatal as possible

The surgical phase began with the extraction of tooth #23. A crestal incision was performed in a very palatal position in order to use the crestal soft tissue in the buccal situation and improve the keratinized soft tissue volume in the buccal position (Figure 9).

**Figure 10:**
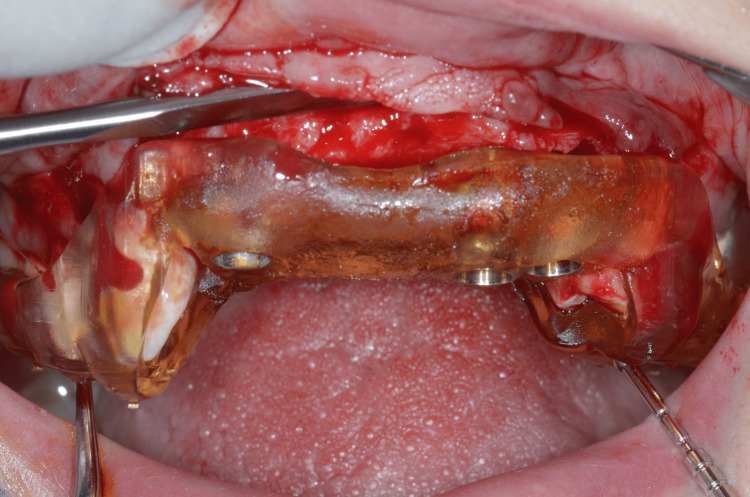
Try-in of the surgical guide

**Figure 11:**
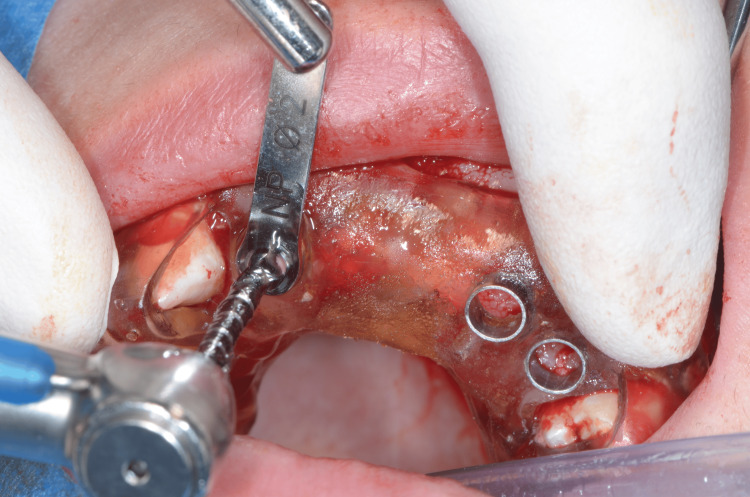
Fully guided osteotomy

**Figure 12:**
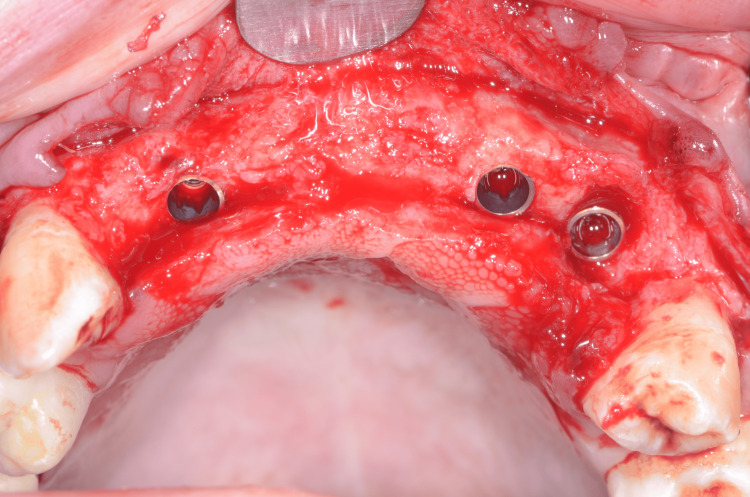
Final positions of implants

The full-thickness flap was lifted, and the tooth-supported stereolithographic guide was tried in (Figure 10). Then, osteotomies were performed using a 2.8-mm-diameter drill to reach the minimum primary stability to achieve an immediate loading (35 N/cm) (Figure 11). Implants (Nobel Active®, Nobel Biocare AB) were placed, and the implants' primary stability allowed an immediate loading (Figure 12).

**Figure 13:**
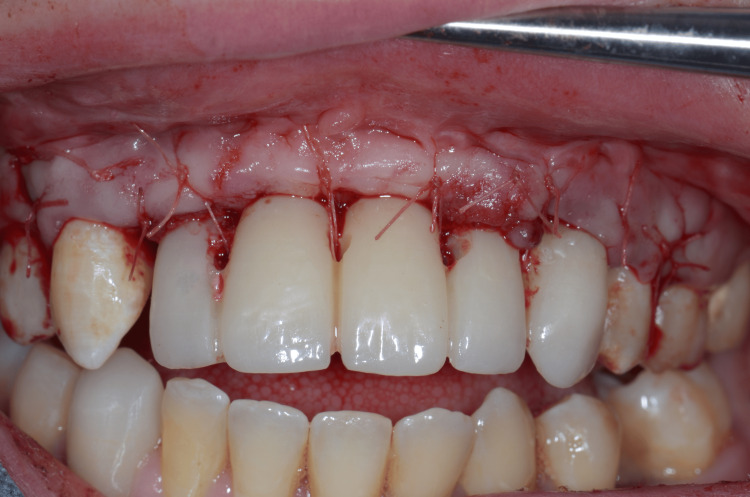
Immediate loading of the temporary screw-retained prosthesis

Provisional abutments were screwed into implants and barely connected with the acrylic resin temporary FPD (Telio CAD®, Ivoclar Vivadent AG) using flowable composite resin (Filtek™ Bulk Fill Flowable Restorative, 3M™, MN, USA). The temporary FPD was unscrewed to design the emerging profile using a chemopolymerizable acrylic resin (Unifast III®, GC Dental, IL, USA). After polishing, provisional FPD was screwed following the manufacturer's recommendations, and screw-access holes were filled using polytetrafluoroethylene and flowable composite resin. Sutures were performed to maintain as coronally as possible the keratinized soft tissue and achieve a prosthetically guided tissue regeneration. Immediate loading of the temporary screw-retained prosthesis was performed (Figure 13).

Intrabuccal radiographs allowed to control implant placement and the good fitting of the temporary FPD into the implants. The patient was reminded postoperative advice. She will be seen again for stitch removal at 10 days, followed by regular follow-up every 15 days for two months.

**Figure 14:**
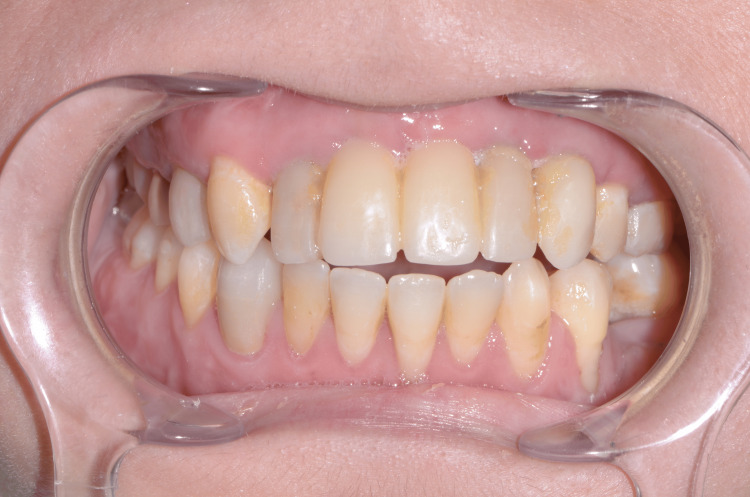
Overall aspect after a four-month healing

**Figure 15:**
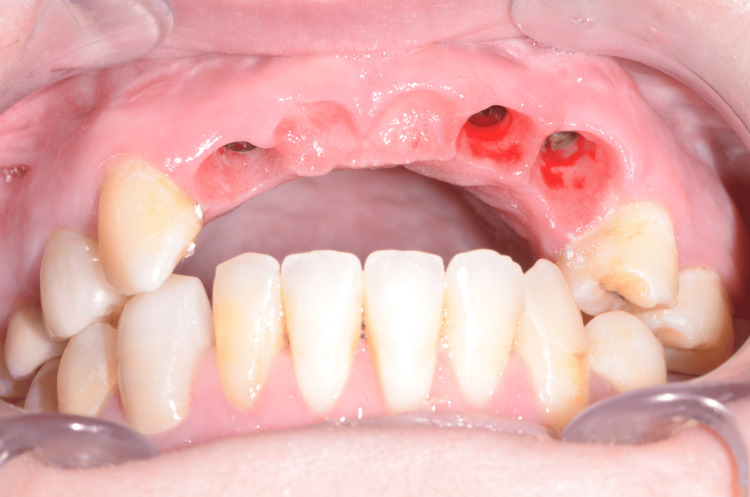
Occlusal view of new papillae after a four-month healing

**Figure 16:**
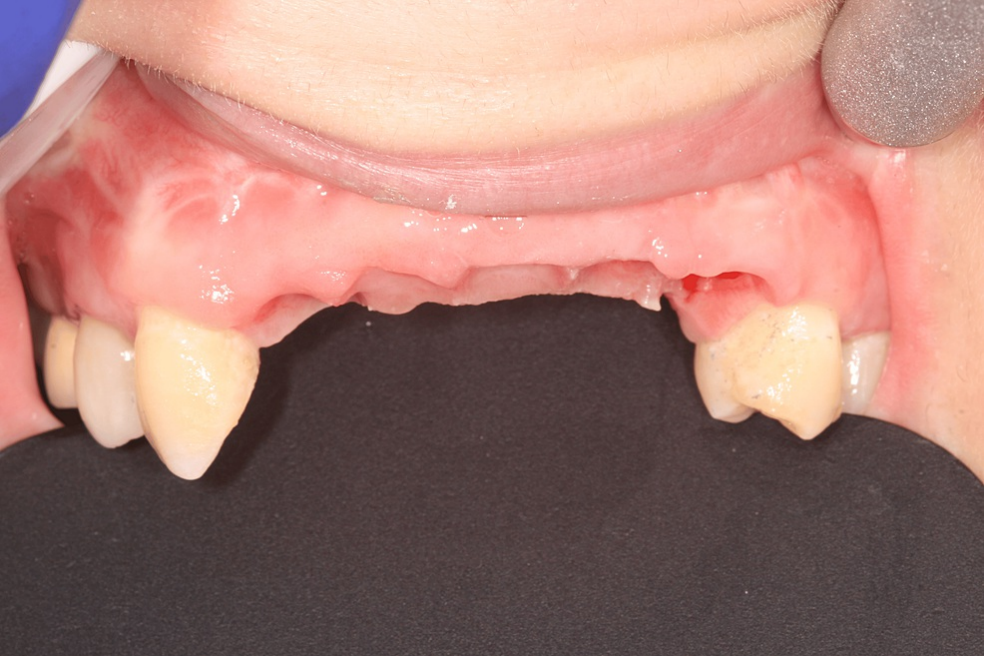
Buccal view of new papillae after a four-month healing

After four months, the prosthetic phase could start. Good soft tissue healing and new papillae could be observed when the temporary FPD was unscrewed (Figures 14-16).

**Figure 17:**
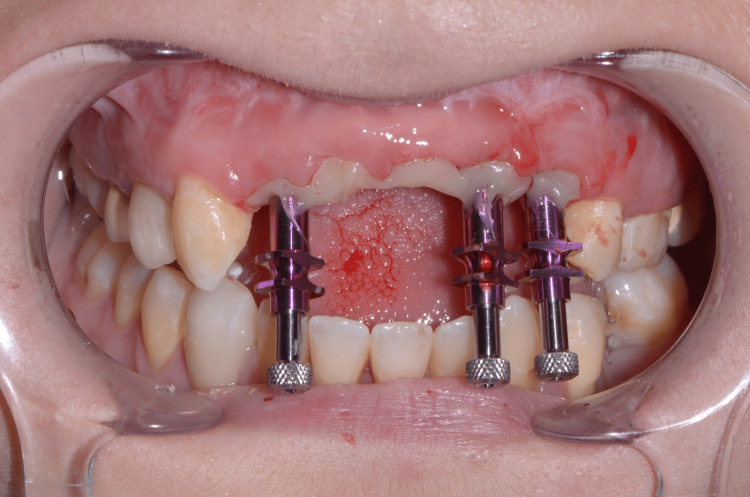
Customized impression copings to transfer gingival shape

**Figure 18:**
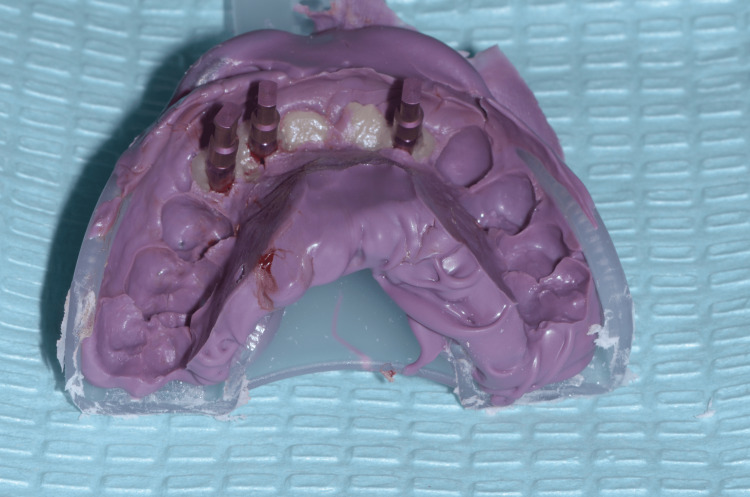
Final impression with recorded emerging profiles

**Figure 19:**
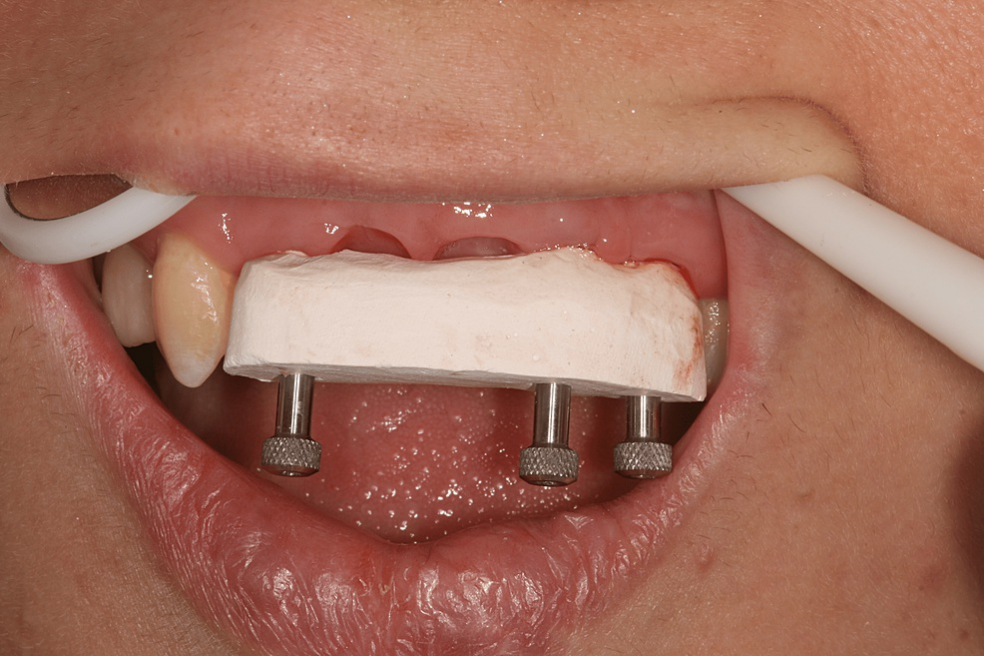
Gypsum validation device

Based on the emerging profile of the temporary prosthesis, customized impression copings have been carried out using flowable composite resin (Filtek™ Bulk Fill Flowable Restorative, 3M™) so that the technician could copy the emerging profile in the definitive FPD (Figure 17) [4]. Conventional impression was made using a standard plastic impression tray (3M™) and a polyether material (Impregum™, 3M™) (Figure 18). A gypsum validation device was used to control the impression accuracy, and maxilla-mandibular relationship was also checked (Figure 19).

**Figure 20:**
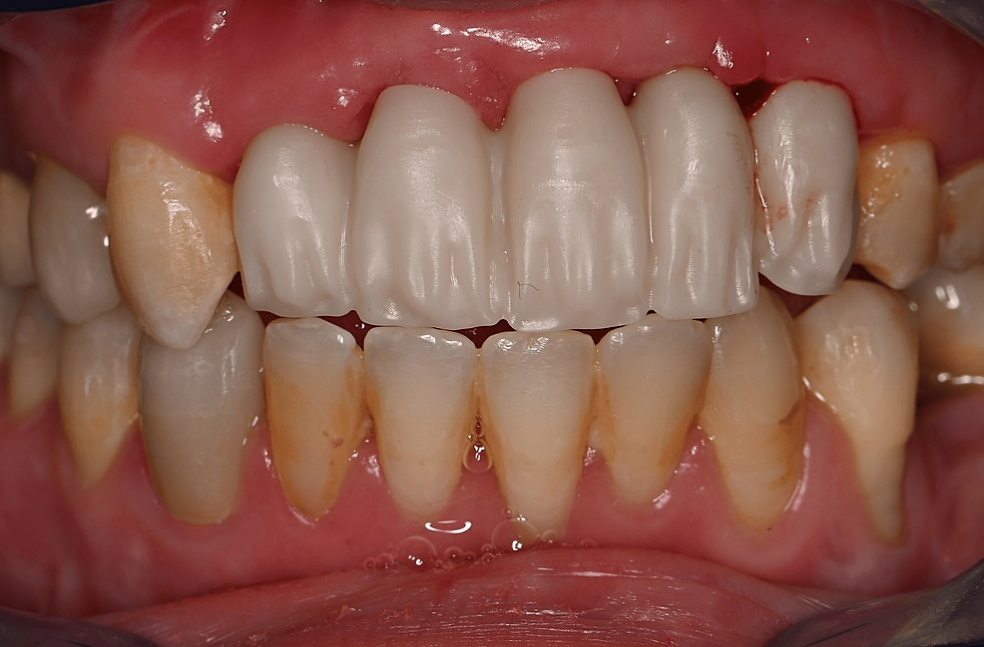
Buccal view of the zirconia framework

**Figure 21:**
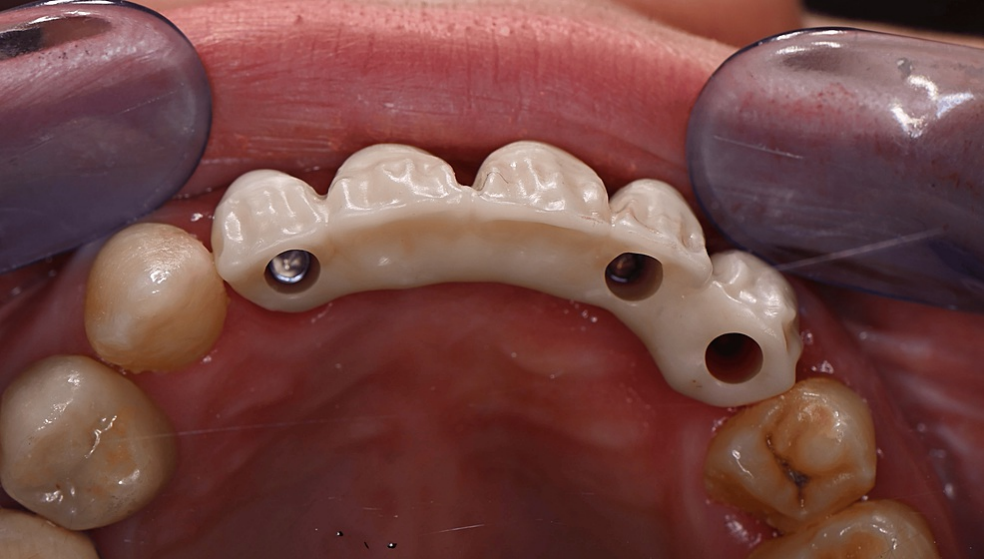
Occlusal view of the zirconia framework

The framework was milled in full zirconia (Prettau 2 Dispersive, A2 shade; Zirkonzahn, South Tyrol, Italy) to meet the esthetic requirements while maintaining good mechanical properties. Polished zirconia only was used in the transgingival areas to prevent any gingival inflammation and ensure good biocompatibility and esthetic results (Figures 20, 21).

**Figure 22:**
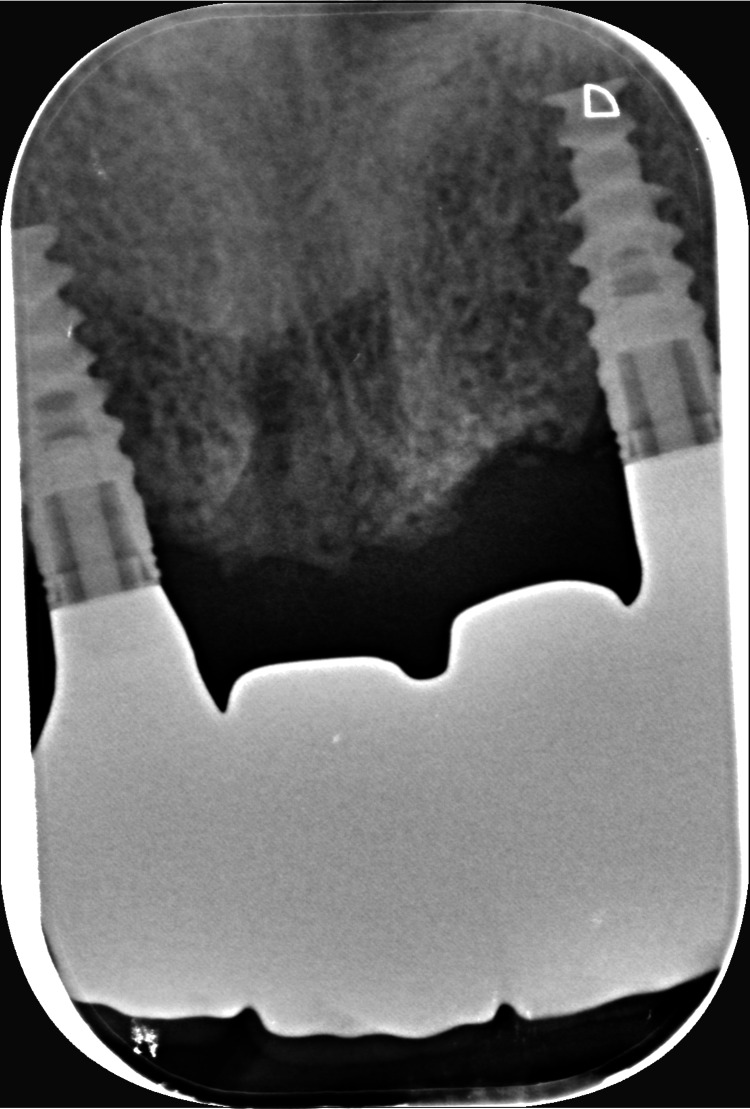
Intraoral radiograph of the framework screwed into implants #12 and #22

**Figure 23:**
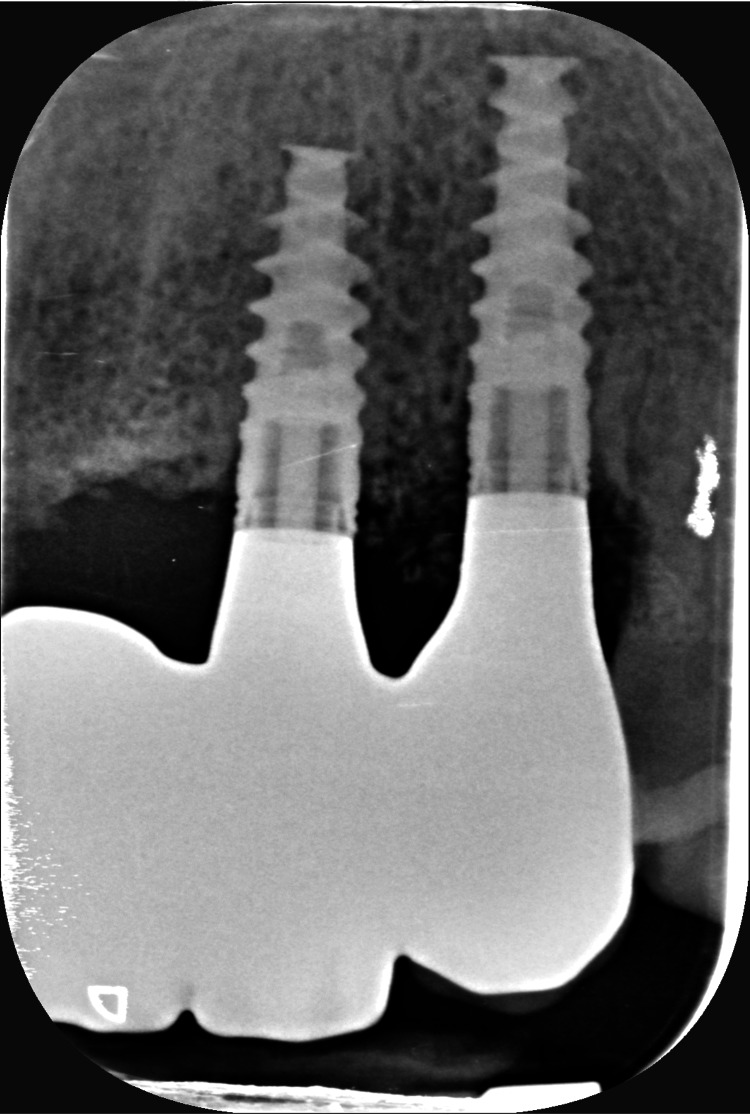
Intraoral radiograph of the framework screwed into implants #22 and #23

Intraoral radiographs were carried out to control the good fitting, and passive tightening should be clinically perceptible (Figures 22, 23). The occlusion is validated, and the fabrication of the cosmetic ceramic is requested. 

**Figure 24:**
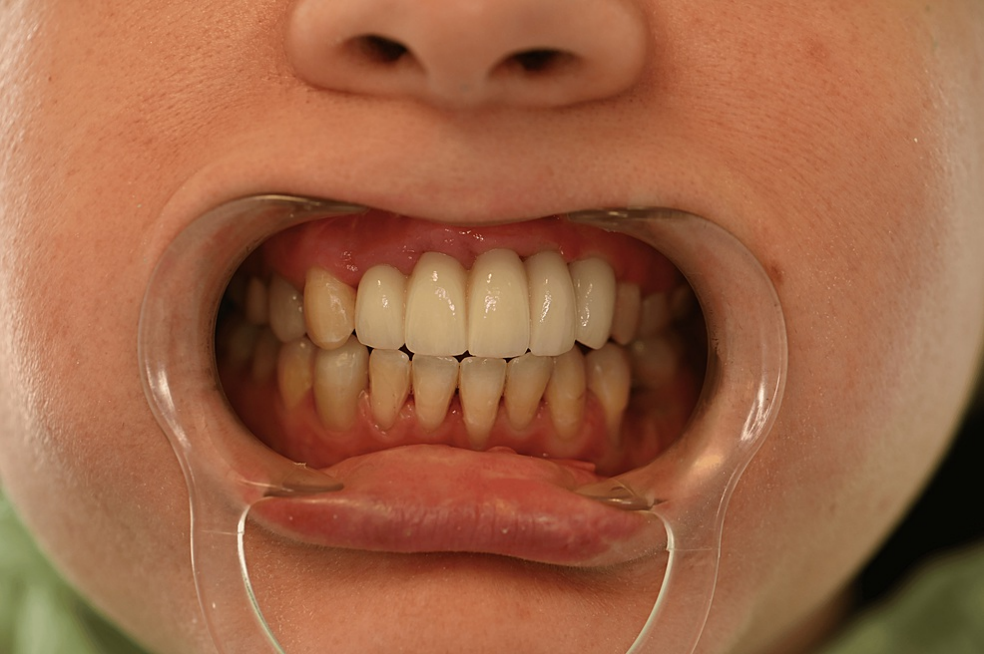
Buccal view of the final prosthetic restoration

**Figure 25:**
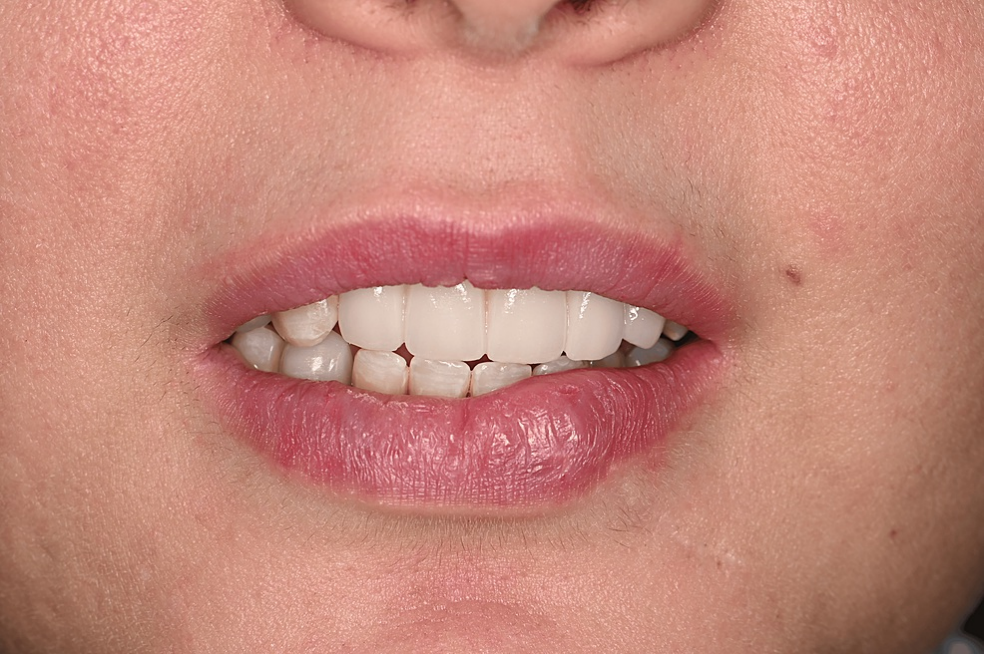
Buccal view of the final prosthetic restoration

The partial FPD was delivered one week later and met the patient's satisfaction. The abutment screws were tightened according to the manufacturer's recommendations. The integration with the patient's smile is acceptable despite the lack of gingival line harmony as expected and discussed with the patient during the initial choice of treatment (Figures 24, 25).

## Discussion

This clinical case showed the overall management of prosthetic rehabilitation in the maxillary esthetic zone in a patient with a very limited mouth opening, from implant planning to delivery of the prosthesis. It also showed soft tissue management to create new papillae in a partially edentulous jaw where prolonged wearing of an RPD has flattened the crestal keratinized mucosa. In implant dentistry, digital technologies (CBCT, IOS, software, etc.) have enabled the dental team to offer patients more comfortable, effective, and less invasive procedures with outcomes that are at least as good as conventional procedures [5].

Fully static guided surgery (sGS) has been used in this clinical situation in line with several studies that reported an improved accuracy of fully sGS compared with freehand surgery, pilot drill sGS, and half sGS [6-8]. However, every step from digital workflow to surgery can generate errors, leading to discrepancies in implant placement [9-11]. Tang et al. also reported that dynamic navigation offers an interesting alternative solution for placing implants in patients with very limited mouth opening as it allows surgeons to work in insufficient spaces [12]. In the current case, a fully static GS has been possible and has enabled the implants to be placed as planned and the temporary screw-retained prosthesis to be delivered on the same day as surgery.

In esthetic areas, pink and white esthetic scores are the gold standard in the objective evaluation of prosthetic restorations [1,2]. In the current situation, the patient did not want a bone augmentation procedure: this was an additional difficulty in the process of reconstructing the papillae. Stefanini et al. reported that soft tissue augmentations displayed stable soft tissue margins without apical displacement in the medium and long term [13]. In addition, Sanz et al. reported the importance of keratinized and attached mucosa on peri-implant health [14]. For this reason, the incision was in the lingual position to use the keratinized crestal tissue to increase the volume of the labial soft tissue. In addition, a connective tissue graft could have been added to this procedure to increase the buccal soft tissue volume, facilitate the appearance of new papillae, and improve the soft tissue margin [15,16]. This soft tissue augmentation could also have been realized after osseointegration, but the patient was satisfied with the esthetic outcome and preferred not to undergo this surgical procedure. The immediate temporary prosthesis was also used to shape peri-implant soft tissue and maintain bone and soft tissue volume [17].

Although it has some limitations such as esthetic properties, monolithic zirconia could meet the biological and mechanical requirements for multiple implant-supported prostheses in different clinical situations [18,19]. In a meta-analysis, Linkevicius and Vaitelis reported that zirconia showed better esthetic outcomes in soft tissue appearance than titanium [20]. According to the literature and given the absence of occlusal stress, a zirconia framework has been used, and esthetic ceramic has been added on the labial face of the framework to compensate for the lack of esthetic properties of this material.

## Conclusions

This clinical case report offered an overview of the use of digital workflow in critical cases. Within the limits of digital technologies, they help the dental team manage complex clinical situations and offer functional and esthetic prosthetic rehabilitation in esthetic areas. Although GS can lead to the expected outcome, there is a learning curve to follow. Nowadays, fully sGS remains the gold standard in implant dentistry to accurately implement the initial prosthetic plan, but dynamic GS and the rise of artificial intelligence could provide new solutions to manage complex clinical situations.
